# Kinematics and Dynamics of Turbulent Bands at Low Reynolds Numbers in Channel Flow

**DOI:** 10.3390/e22101167

**Published:** 2020-10-16

**Authors:** Xiangkai Xiao, Baofang Song

**Affiliations:** Center for Applied Mathematics, Tianjin University, Tianjin 300072, China; xiaoxk2018@tju.edu.cn

**Keywords:** turbulent bands, obliqueness, advection speed, wave generation, inflectional instability

## Abstract

Channel flow turbulence exhibits interesting spatiotemporal complexities at transitional Reynolds numbers. In this paper, we investigated some aspects of the kinematics and dynamics of fully localized turbulent bands in large flow domains. We discussed the recent advancement in the understanding of the wave-generation at the downstream end of fully localized bands. Based on the discussion, we proposed a possible mechanism for the tilt direction selection. We measured the propagation speed of the downstream end and the advection speed of the low-speed streaks in the bulk of turbulent bands at various Reynolds numbers. Instead of measuring the tilt angle by treating an entire band as a tilted object as in prior studies, we proposed that, from the point of view of the formation and growth of turbulent bands, the tilt angle should be determined by the relative speed between the downstream end and the streaks in the bulk. We obtained a good agreement between our calculation of the tilt angle and the reported results in the literature at relatively low Reynolds numbers.

## 1. Introduction

Much below the linear critical Reynolds number of the parabolic channel flow, transition to turbulence can occur under finite-amplitude perturbations, i.e., via a subcritical transition. Numerous studies have established that turbulence takes the form of discrete turbulent bands that are oblique to the streamwise direction, interspersed with laminar flow, at transitional Reynolds numbers [1,2,3,4,5,6,7,8,9,10]. Similar banded turbulent structures have also been observed in other quasi-two-dimensional flows, i.e., systems with one confined dimension and two extended dimensions, such as plane Couette [11,12,13], Taylor Couette [14,15], annular pipe [16] and Wallefe flows [17]. Therefore, the coexistence of laminar and turbulent states in the form of banded turbulent structures is a common feature of turbulence at transitional Reynolds numbers of a broad variety of shear flows. Recent investigations into these structures have greatly advanced the understanding of the subcritical transition in these flows [10,18]. In the following discussion, for channel flow, the streamwise, wall-normal and spanwise directions are denoted as *x*, *y* and *z*, respectively, time is denoted as *t* and the half-channel-height as *h*. The flow is assumed driven by a constant volume flux and the Reynolds number is defined as $Re=\frac{U_{c}h}{\nu }$, where $U_{c}$ is the centerline velocity of the unperturbed parabolic flow and ν the kinematic viscosity of the fluid.

The first observation and many numerical studies of turbulent bands in channel flow were performed by numerical simulations in relatively small computational domains, either normal or tilted, in which the structure, kinematics and dynamics of turbulent bands are rather restrained [1,2,4,19,20]. Particularly, narrow tilted domains force turbulent bands to be parallel to the narrow edge, which practically assumes infinitely long bands in combination with periodic boundary conditions. Nevertheless, this greatly reduces the computational cost and allows studying the kinematics and dynamics of bands over large time scales [9,20] and offers conveniences for studying the mean flow and wavelength of the band pattern [4,10]. In a domain tilted by $24^{\circ }$, Tuckerman et al. [4] reported that turbulent bands propagate approximately at the bulk speed of the flow, with a slight decreasing trend with the Reynolds number (the speed crosses the bulk speed at Re≃1100). In a similar approach as Avila et al. [21] for pipe flow and Shi et al. [22] for plane Couette flow, Gomé et al. [20] also showed finite lifetime and splitting nature of bands and determined the on-set of sustained turbulence in channel flow to be at Re≈950 by balancing the super-exponential decay and splitting processes, in a domain also tilted by $24^{\circ }$. The subcritical transition to turbulence in plane Couette flow in tilted domains has been concluded to fall in the universality class of directed percolation [23] and the work of Gomé et al. [20] seems to suggest the same transition scenario in channel flow. However, the imposed tilt angle of the domain seems to affect the statistical results. For example, the simulations in a domain tilted by $45^{\circ }$ [9] showed very different lifetimes of bands from the results of Gomé et al. [20]. Specifically, the former reported that turbulent bands are sustained at Re>620, whereas the latter suggested that in fact the lifetime stays finite and is below 200 time units at Re<700. The effect of the imposed tilt angle has not been thoroughly investigated. Besides, the usual narrow tilted domain only allows multiple bands to form parallel band pattern, i.e., bands are forced to take the same orientation.

Large domains pose a lesser restriction on turbulent bands. In recent years, a few studies have been dedicated to turbulent bands in large normal domains in experiments [3,9] and simulations [5,6,7,8,24,25]. If the domain is large enough, given a proper localized perturbation, turbulence elongates obliquely with respect to the streamwise direction and forms a fully localized band (localized both in its length direction and in its width direction). The existence of the two ends of the band adds further complexity to the flow. Paranjape [9] reported in experiment that at Re<660, a turbulent band shrinks and will decay so that the flow will relaminarize in the end, because the growth at the downstream end (referred to as the head hereafter) is slower than the decay at the upstream end (referred to as the tail hereafter). At higher Reynolds numbers, a turbulent band becomes sustained because the growth at the head outperforms the decay at the tail and will grow in length. Numerical studies [6,7,24] agree with the experiments. Therefore, it has been confirmed that the growth of a band is unidirectional, driven by the head [7,8,9,24]. Because streaks decay at the tail and are generated at the head, an individual band undergoes a spanwise shift as a whole, aside from being advected in the streamwise direction. Shimizu and Manneville [8] mentioned that the spanwise drift speed is 0.1 and Xiao and Song [24] reported a close value of 0.08. Noticing the periodic streak generation at the head, Kanazawa [7] and Xiao and Song [24], respectively, proposed mechanisms behind the wave generation at the head, which are discussed in more detail below.

In fact, it was found that the length of a band does not grow infinitely. The length ‘at equilibrium’ of a band at Re=660 was shown to be about 300 *h* and the length seems to increase with Re [7]. As the length is sufficiently large, the fast decay of the tail limits the growth, and splitting may occur with a daughter band nucleated. At relatively low Reynolds numbers, the splitting is longitudinal, i.e., the daughter band is parallel to the mother band. As Reynolds number increases (Re≳800), transverse splitting (or branching) can also occur, nucleating daughter bands with the opposite tilt direction such that the flow pattern becomes two-sided (the criss-cross pattern) [8,9]. However, the study of the splitting of bands and the underlying mechanism is still rare.

In the presence of multiple bands, given that bands have a spanwise shift speed as a whole and can grow in length, close bands with opposite orientations may collide. Even parallel bands, when located sufficiently close to each other, were shown to interact also [6,8]. The dynamics of individual bands and the interaction between bands determine the pattern that bands can form and therefore, determine the statistical aspect of the transition to turbulence [8]. Using unprecedented large domain and simulating up to very large times (up to $O(10^{5})$ time units), Shimizu and Manneville [8] showed that turbulent bands can only form one-sided (parallel) pattern at low Reynolds numbers (Re≲924), breaking the spanwise symmetry, which is restored only at higher Reynolds numbers. Directed percolation was found to reasonably well describe the transition process toward featureless turbulence at further higher Reynolds numbers, as also proposed by Sano and Tamai [26] in experiments. Interestingly, the one-sided pattern of turbulent bands at lowest Reynolds numbers seems to justify the use of tilted domain in which bands are forced to be parallel, although the tilt angle was shown about $40^{\circ }$–$45^{\circ }$ below Re≃900 [6,7,8,9] rather than $24^{\circ }$ as used in some studies [4,20].

Although a great advancement in the understanding of turbulent bands has been made in recent studies, many problems even for individual turbulent bands have not been well understood, for example the mechanisms underlying the growth of bands at the head and the decay at the tail, the tilt angle selection and the self-sustaining mechanism of the bulk of turbulent bands. We discuss some of these problems in this paper.

## 2. The Head

### 2.1. Propagation Speed of the Head

**Figure 1:**
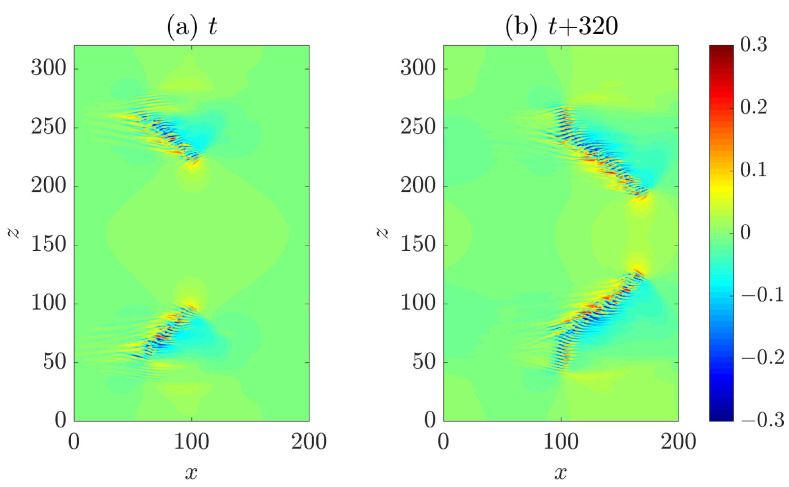
Turbulent bands with different orientations at Re=750. (**a**,**b**) The streamwise direction is in the positive *x* direction and *z* denotes the spanwise direction. Streamwise velocity fluctuations in the *x*-*z* cut plane at y=−0.5 are plotted as the colormap with blue representing low speeds and red representing high speeds compared to the basic flow. The two panels are separated by 320 time units.

Firstly, we investigated the advection speed of the head. It has been reported that the head of a turbulent band, which is always located at the downstream end, propagates in both streamwise and spanwise directions [6,8,24]. The spanwise motion can be in either positive or negative spanwise direction and the specific direction is correlated with the orientation of the band (see Figure 1). The head of the upper band moves downward (in negative spanwise direction) while that of the lower band moves upward, given their opposite orientations. Bands with similar orientation as the upper one are referred to as right-going bands, and those with the opposite orientation are referred to as left-going bands. This correlation can be intuitively understood because the head continually generates turbulence by invading laminar flow region on one side. We revisit this point in Section 2.2. Xiao and Song [24] measured the speeds at Re=750 by tracking the head and reported a streamwise speed of $c_{x}=0.85$ and a spanwise speed of $c_{z}=0.1$ (absolute value).

To investigate the Re-dependence of the speeds and also for calculating the tilt angle of turbulent bands in Section 4, we measured the speeds in the low Reynolds number regime ranging from Re=670, which is nearly the lowest Reynolds number for sustained bands, to Re=1050 at which frequent splitting and branching of bands were reported to occur [8,9]. For this study, the Reynolds numbers, domain sizes and resolutions are listed in Table 1. It has been shown that, at Re=660, a band can continuously grow up to the length of approximately 300 *h* [7]. The length can be much larger at higher Reynolds numbers [7,8]. The domain sizes used in our study are not large enough for the band to reach the length ‘at equilibrium’, rather we only require the domain size to offer sufficiently long time for the head to reach its characteristic propagation speed. The simulation was stopped when the head and tail were too close to each other and started to interact due to the periodic boundary conditions. Xiao and Song [24] already showed that the speed of the head of turbulent bands at Re=750 is not affected by the domain size by comparing the speeds measured in domains with $L_{x}=L_{z}=120$ *h* and $L_{x}=L_{z}=320$ *h*.

**Figure 2:**
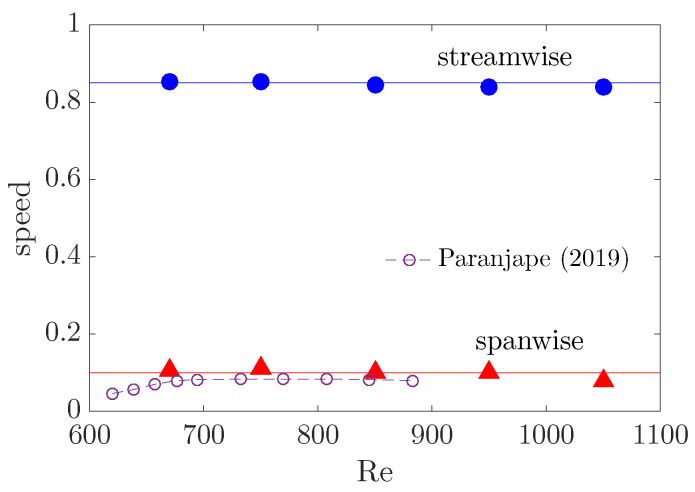
The streamwise (circles) and spanwise (triangles) speed of the head of turbulent bands at various Reynolds numbers. Note that it is the absolute value of the spanwise speed plotted given that the speed can take either positive or negative values. The two solid lines at 0.85 and 0.1 are plotted to guide the eyes. The experimental measurement of the spanwise speed [9] is plotted as the dashed-circle line for comparison.

At each Reynolds number, we generated a fully localized turbulent band directly at low Reynolds numbers using the method proposed by Song and Xiao [25]. After the band has sufficiently developed, the head was tracked over a time window of O(500) time units and the average speed was calculated based on the position and time separation. The results in Figure 2 show that both the streamwise and spanwise speeds stay nearly constant for all Reynolds numbers investigated, at 0.85 and 0.1, respectively. Besides, the speeds were shown to be rather stable, i.e., only fluctuate slightly in time around the respective averaged values for Re=750 [24], which is also the case for other Reynolds numbers in this study.

In experiments, Paranjape [9] showed that the spanwise speed of the head slowly decreases from 0.085 at Re≃700 to 0.08 when the Reynolds number is increased to Re≃850 (see the dashed-circle line in Figure 2). Besides, Paranjape [9] reported a streamwise speed of the entire band of about 0.75 between Re=670 and 900, but did not report the streamwise speed of the head. They also reported the speeds between Re=600 and 670, in which regime we could not obtain a sustained turbulent band in our DNS. It can be seen that our spanwise speed is systematically larger than the experimental measurement [9] (see Figure 2). The difference could possibly be attributed to the periodic boundary condition used in our numerical simulations, although Xiao and Song [24] mentioned that the $L_{x}=L_{z}=120$ *h* box gives the same speed as that given by the $L_{x}=L_{z}=320$ *h* box at Re=750. It may equally be attributed to the side-wall effect in experiments. Simulations in much larger periodic boxes or in a channel with side walls are needed to confirm about this point. Nevertheless, the two sets of speeds are close to each other.

### 2.2. Wave Generation at the Head and the Tilt Direction of the Band

**Figure 3:**
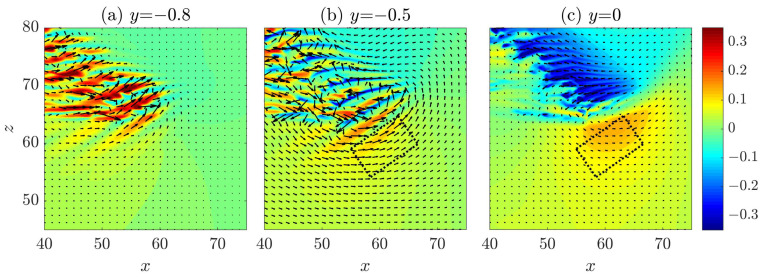
Large-scale circulation flow in the neighborhood of the head. Streamwise velocity is plotted as the colormap in the *x*-*z* cut plane at: y=−0.8 (**a**); y=−0.5 (**b**); and y=0 (**c**). In each panel, the in-plane velocities are plotted as vectors. The dotted rectangle (size 6 *h* × 10 *h*) marks approximately the area where the first visible wave that is continually generated at the head in the frame of reference co-moving with the head.

In this section, firstly we discuss about some recent studies on the dynamics of the head. Therefore, a part of the results shown below is not original. It has been noticed that the head drives the growth of turbulent bands by continually generating waves, in the form of alternating high- and low-speed streaks and arrays of vortices, while moving into the adjacent laminar region [7,8,9,24]. Figure 3 shows the wave-like structure of the head. Contours of streamwise velocity fluctuation are plotted in the *x*-*z* plane at y=−0.8 (close to the wall, see Figure 3a), at y=−0.5 (Figure 3b) and in the mid-plane y=0 (see Figure 3c). It can be seen that the flow is characterized by high-speed streaks close to the wall. In the mid-plane, the flow is characterized by low-speed streaks in the bulk, which almost merge and form a connected low-speed region, and is characterized by a high speed region at the head (see the yellow spot in Figure 3c). At y=−0.5, the flow exhibits wave-like alternating low and high-speed streaks. The large-scale (compared with the wave-like streaky structures) flow in the neighborhood of the head manifests a circulation (see [6,24]), which is counter-clockwise for a right-going band as shown. Duguet and Schlatter [27] proposed a mechanism for the formation of large-scale flow around turbulent bands in plane-shear flows. Their theory applies to the large-scale flow associated with the bulk region of the band and describes the band as the advection of small-scale structures (streaks) by the large-scale flow. However, they did not explicitly study the large-scale flow at the head.

**Figure 4:**
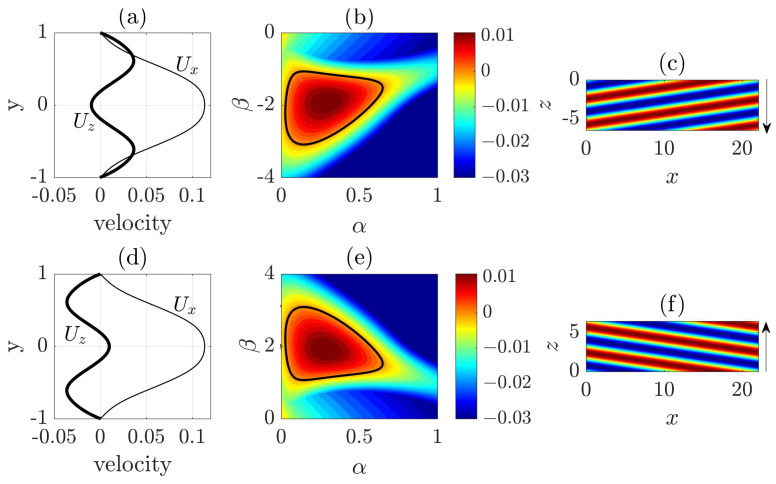
Linear instability of the modified velocity profile at the head that is spatially and temporally averaged in the rectangle shown in Figure 3b: left-going bands (**a**–**c**); and right-going bands (**d**,**e**). (**a**,**d**) Velocity profiles. Wall-normal component is very small and neglected; (**b**,**d**) The maximum eigenvalue in the wave number plane, in which α is the streamwise wave number and β the spanwise wavenumber. The bold line marks the neutral stability curve; (**c**,**f**) Contours of streamwise velocity of the most unstable disturbances at the cut plane y=−0.5. Red and blue colors represent high speed and low speed regions, respectively. The arrows show the direction of the spanwise wave speed. Similar analysis for the modified velocity profiles averaged in different regions were reported in [24,25].

The dotted rectangle in Figure 3 marks the approximate region in which the first visible high speed streak is periodically generated. The vector plot of the in-plane velocities shows that, at y=−0.5 (see Figure 3b), the vectors in the rectangle overall point to the positive *z* direction, and, at y=0 (see Figure 3c), the vectors overall point to the negative *z* direction. This hints that there should be an inflection in the spanwise velocity profile in this region, which may be inflectionally unstable. Based on this observation, Xiao and Song [24] investigated the local mean flow at the head and attributed the wave generation at the head to an inflectional instability associated with the modified local mean flow. For the ease of discussion, we measured the averaged velocity profiles at the head again in a different region and for a different turbulent band compared to those reported in [24] (see Figure 4). Both streamwise and spanwise velocity profile (the parabolic base flow is not included) show inflection. These profiles are measured at a right-going band similar to the upper one in Figure 1 and the one shown in Figure 3. Figure 4b shows the unstable region in the wavenumer plane (the region enclosed by the bold line) and Figure 4c shows the streaky flow pattern of the most unstable disturbance (see also [24,25]). It can be seen that these streaks are tilted about the streamwise direction and the tilt direction is the same as the waves that can be seen at the head of right-going bands in Figure 1 and Figure 3. Besides, the most unstable wave move downward, i.e., in the negative spanwise direction (see the arrow), just as the head of the right-going band. By the symmetry of channel flow about the *x*-*y* plane, it can be inferred that the velocity profiles at the head of a left-going band will be similar to those shown in Figure 4d, with the sign of the spanwise velocity changed. We performed a similar linear analysis here and show the unstable region in the wavenumber plane in Figure 4e and the most unstable disturbance in Figure 4f. Clearly, we can see a spanwise symmetry in the distribution of eigenvalues and in the flow pattern by comparing to Figure 4b,c. The waves shown in Figure 4f are tilted in the opposite direction compared with the waves in Figure 4c and move in the positive spanwise direction, which is consistent with the structure and kinematics of the head of a left-going band. In a word, linear stability analysis gives qualitatively similar flow structures and kinematics as that of the head. The nonlinear development of disturbances was shown to give similar flow structures as those at the head [24]. Therefore, Xiao and Song [24] proposed that the growth of turbulent bands is driven by the inflectional instability locally at the head. Further, Song and Xiao [25] performed a non-modal analysis of the inflectional velocity profiles and showed an Orr-mechanism via which disturbances can achieve a fast growth in energy at the early stage (by a factor of 100 within about 15 time units for Re=750). Subsequently, the modal instability takes part and starts to dominate the growth at later points of time. The linear instability together with the fast non-normal growth at the early stage are able to result in a fast growth of the unstable waves at the head. Reaching a certain amplitude, the waves become turbulent when nonlinearity sets in and subsequently evolve inside the bulk of the band in the form of streaks and vortices.

Based on these discussions, here we propose that the moving direction and the tilt direction of a band are probably determined by what type of local flow is formed when a localized perturbation is introduced: One similar to that shown in Figure 4a generates a right-going band and one similar to that shown in Figure 4d generates a left-going band. In fact, the technique proposed by Song and Xiao [25], with which we generated the bands in Figure 1, is based on this mechanism. The key of the technique is to impose a localized body force that moves with the speed of the head and induces a locally inflectional flow. It can offer a control on the tilt direction of the generated bands because it offers a control on the spanwise velocity profile (to be similar to either the one in Figure 4a or the one in Figure 4c) and on the moving direction of the force. The efficacy of the technique in turn supports that some key characteristics of a band are determined by the local inflectional mean flow at the head.

Although the linear instability, as well as the non-normality, associated with the local mean flow seem to be the mechanism underlying the wave generation and growth of turbulent bands, how this inflectional local mean flow is formed and sustained is still not sufficiently understood. Tao et al. [6] observed that, when the computational domain is too small, a band may interact with its periodic image and decay. Based on this observation, they proposed that the sustainment of a turbulent band relies on the secondary large-scale flow surrounding the band, and a close neighbor may affect this large-scale flow and eliminate the band. Given that a turbulent band is driven by the head, this observation seems to imply that the head of a band is sustained by the large-scale flow, see Figure 3. However, Kanazawa [7] proposed a completely different scenario. They added a damping term to the Navier–Stokes equations, using which they suppressed the formation of the body of a band and isolated the head, and observed that the head can be self-sustained as a nonlinear periodic orbit. This periodic orbit is characterized by an array of streaks and vortex tubes that resemble the flow structure at the head. Because the band does not form under the damping, the large-scale flow is also absent, although there is still a local circulation flow associated with the localized periodic orbit itself. This seems to contradict the conclusion of Tao et al. [6] that a band relies on the large-scale flow surrounding the band. Further, Kanazawa [7] studied the bifurcation of the periodic orbit in the damped system and reported a saddle-node bifurcation that gives rise to the periodic orbit. Below the saddle-node bifurcation point, no such exact coherent structures exist. Therefore, the authors proposed that this self-sustained periodic orbit and the subsequent bifurcations to torus and chaos is responsible for the formation and sustaining mechanism of turbulent bands. However, they failed to obtain a periodic orbit and reproduce the bifurcations as the damping parameter vanishes, i.e., in the Navier–Stokes equations without an artificial damping. Obtaining such a periodic solution may finally elucidate the appearance and self-sustaining mechanism of fully localized turbulent bands [7].

Kanazawa [7] did not show why and how exactly this periodic orbit generates wave-like streaks or vortices, rather, only described them as the characteristics of the periodic orbit. In fact, the inflectional instability proposed by Xiao and Song [24] may be related to this periodic orbit. The possible connection is that the circulation associated with the periodic orbit may be locally inflectional and responsible for the wave generation. The inflectional profiles of Xiao and Song [24] are just temporal-spatial averages at the head and only depend on *y*. The averaging leaves out the streamwise and spanwise dependence of the real local flow at the head; therefore, Xiao and Song [24] pointed out that this may be why their stability analysis cannot quantitatively capture some characteristics of the waves at the head, such as the value of the tilt angle of the waves with respect to the streamwise direction. The analysis of this three-dimensional periodic orbit may be needed to more quantitatively understand the dynamics of the head.

## 3. The Bulk

The bulk of a turbulent band is defined as the elongated part that is sufficiently far from the head and tail, which does not significantly vary on large-scale and can be considered to be at an ‘equilibrium state’.

### 3.1. The Flow Structure

**Figure 5:**
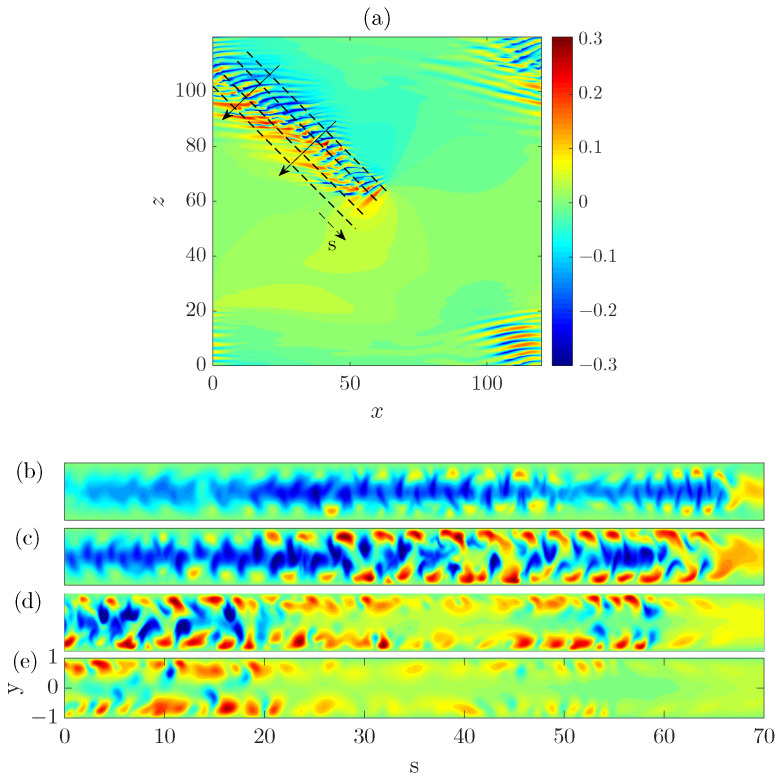
(**a**) Streamwise velocity fluctuations of a band at Re=750 plotted in the *x*-*z* plane at y=−0.5. The four dashed lines mark the positions of four cut planes perpendicular to the wall, in which streamwise velocity fluctuations are plotted in (**b**–**e**). The *s*-axis goes from top-left to bottom-right along the lines. The two arrows, at s=10 and 40, respectively, show the sequence of (**b**–**e**). The length in *y* direction is stretched by a factor of 3.

Many studies have noticed the wave-like form of the bulk of turbulent bands [6,7,9,24], i.e., regularly aligned and distributed streaks along the band. In Figure 5a, the streamwise velocity fluctuations are plotted as the colormap in the *x*-*z* plane at y=−0.5 (blue color shows low speed and red shows high speed region). Low-speed streaks (blue) are nearly parallel to the streamwise direction and show nearly a periodic pattern. On the upstream edge, high-speed streaks (red) can be observed but do not show a strong periodic pattern as the low-speed streaks. It should be noted that the tilt angle, with respect to the streamwise direction, of the steaks in the bulk is significantly lower than that at the head. Both low speed and high-speed streaks are nearly parallel to the streamwise direction. However, it still can be noticed that these two groups of streaks exhibit opposite tilt directions. The four dashed lines mark the positions of four cut planes perpendicular to the wall, in which streamwise velocity fluctuations are plotted to visualize the structure of the band in the wall-normal direction (see Figure 5b–e). A two-layer structure can be observed, which can be expected from the symmetry of the base flow about the channel center-plane. Each layer consists of staggered high- and low-speed streaks, and, in each layer, high-speed streaks are located near the wall and low-speed streaks near the channel center-plane. Figure 5e and the part between s=20 and 50 in Figure 5d show that, on the upstream, high-speed streaks are the dominate structures. On the downstream, low-speed streaks dominate (see Figure 5b and the part between s=0 and 20 in Figure 5c). In between, high-speed and low-speed streaks are comparable (see the part between s=20 and 50 in Figure 5c and between s=0 and 20 in Figure 5d), and this is the most energetic and turbulent region.

**Figure 6:**
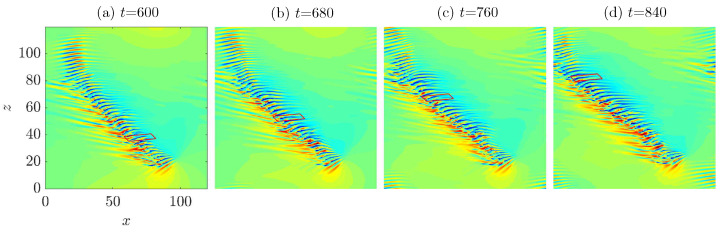
Tracking of a low-speed streak (enclosed by a parallelogram) at Re=750 in the frame of reference co-moving with the head. (**a**) t=600; (**b**) t=680; (**c**) t=760; (**d**) t=840.

Xiao and Song [24] showed that the generated streaks move away from the head in the frame of reference co-moving with the head, and that streaks decay at the tail of the band. To show this process explicitly, we selected a low-speed streak and tracked it (see Figure 6). The tracking lasted for hundreds of time units until the streak reaches the tail of the band, without a significant change in the shape of the streak.

### 3.2. Advection Speed of the Streaks inside the Bulk

Next, we quantitatively studied the advection speed of the streaks. The advection speed can be estimated by tracking an individual low-speed streak, as shown in Figure 6. Alternatively, it is possible to measure the speed of an array of streaks as a whole. We adopted the latter approach. We used the velocity data on the cut plane of y=−0.5, which well cuts through the streaks and offers a nearly optimal visualization of the flow pattern (see Figure 5). Nevertheless, a cut plane close to the wall, which would cut through high-speed streaks that are located close to the wall (see Figure 5), is equally applicable. We used the Structural Similarity Index Measure (SSIM) method [28] from image processing, which accesses the similarity between two images based on luminance, contrast and structure of the images. The method is detailed in Section 6.

**Figure 7:**
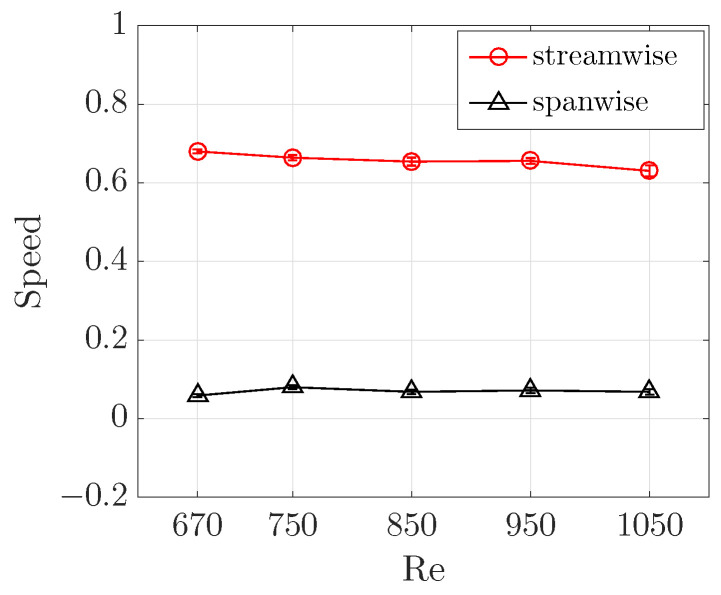
Advection speed of the low-speed streaks at a few Reynolds numbers. The spanwise speeds are $c_{z}=0.068$, 0.076, 0.069, 0.071 and 0.068, and the streamwise speeds are $c_{x}=0.68$, 0.66, 0.65, 0.65 and 0.63, for Re=670, 750, 850, 950 and 1050, respectively.

The advection speed of low-speed streaks for a few Reynolds numbers are shown in Figure 7. The results show that, in the low Reynolds number regime between 670 and 1050, the streamwise advection speed slowly decreases from 0.68 to 0.63, whereas the spanwise speed seems to stay nearly constant at around 0.07. Note that the streamwise speed is very close to the bulk speed of the flow, which is 0.67. In fact, in Figure 6, the parallelogram was moved at the speeds we measured in this way and very well tracked the streak over hundreds of time units. Paranjape [9] reported that the phase speeds of the exact nonlinear traveling wave solution they obtained at Re=720 are $c_{x}$ = 0.77 and $c_{z}=0.06$, which are close to our results, suggesting a strong connection between their traveling wave solution and turbulent bands.

## 4. Tilt Angle of Turbulent Bands

The tilt angle of turbulent bands at Re<1000 was reported in experiments by Paranjape [9]. Their measurements showed that the angle stays nearly constant close to $45^{\circ }$ below Re≃900 and decreases to approximately $30^{\circ }$ above Re=950. The decreasing trend was also reported by Shimizu and Manneville [8]. A few numerical studies also reported the tilt angle at some Reynolds numbers; for example, Kanazawa [7] reported $41^{\circ }$ at Re=660, Tao et al. [6] reported approximately $40^{\circ }$ at Re=700 and Xiao and Song [24] reported an angle of about $39^{\circ }$ at Re=750, which are lower than but close to the experimental results of Paranjape [9]. The small difference may be attributed to the periodic boundary condition used in simulations and to the specific methods of quantifying the tilt angle.

However, the mechanism underlying the tilt angle selection is still not well-understood. Prior studies simply measured the tilt angle by considering the entire band as a tilted object based on image processing or in similar manners [6,9]. Differently, here we propose that the tilt angle should be more fundamentally determined by the propagation speed of the head and the advection speed of the streaks inside the bulk. More specifically, the speed of the streaks inside the bulk relative to the head should determine the tilt angle of the band. Based on our measurements shown in Figure 2 and Figure 7, we calculated the tilt angle of the band as

(1) $$\theta =\arctan\frac{|c_{z,\mathrm{streak}}-c_{z,\mathrm{head}}|}{|c_{x,\mathrm{streak}}-c_{x,\mathrm{head}}|}.$$

**Figure 8:**
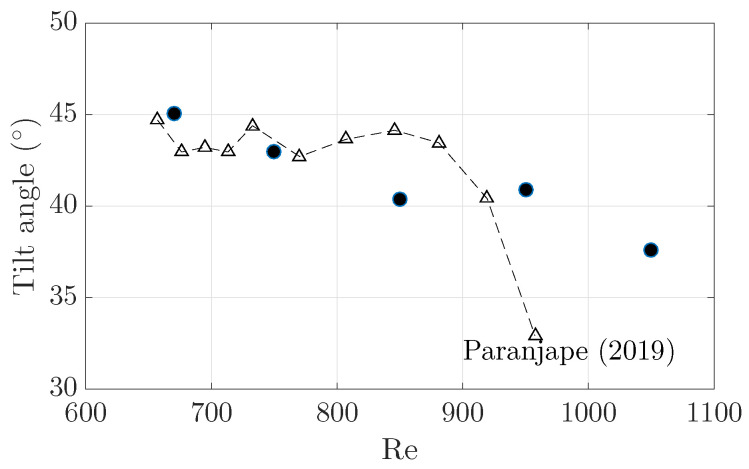
The tilt angle of turbulent bands, calculated with Equation (1), at a few Reynolds numbers. The experimental measurements of Paranjape [9] are plotted as the dashed-triangle line for comparison.

The result is shown in Figure 8. Our calculations agree well with the experimental result of Paranjape [9] below Re≃900. However, at Re=1050, our calculation appears to be much higher than their measurement: our calculation gives $37^{\circ }$ for Re=1050, whereas it was estimated to be around $30^{\circ }$ in experiments. Nevertheless, our calculation gives the decreasing trend in the tilt angle as Re is increased to around Re=1000 and above.

**Figure 9:**
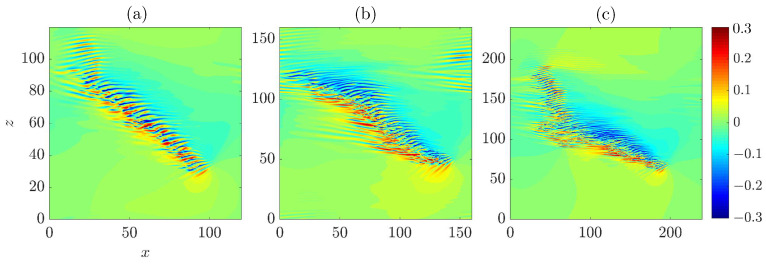
Turbulent bands at: Re=670 (**a**); Re=950 (**b**); and Re=1050 (**c**).

The possible reason for the significant difference between our calculation and the experimental measurements at Re=1050 can possibly be understood by inspecting the structure of the band as Re increases (see Figure 9). We can see that, at Re=670, the band has a well-defined banded structure, i.e., the width (e.g., the streamwise extension) of the band does not significantly change along the band (see Figure 9a). At Re=950, the tail of the band seems to broaden and the width of the band may not be constant along the length direction any more (see Figure 9b). Further at Re=1050, the band significantly delocalizes: The bulk broadens gradually towards the tail and part of the band turns into an extended turbulent area (see Figure 9c). By image processing the entire band, as in the measurements of Paranjape [9] and Tao et al. [6], the calculated tilt angle at Re=1050 will certainly be smaller than our calculation that is only based on the information of the low-speed streaks and the head. This disagreement will be small at low Reynolds numbers when turbulence is well-banded.

The agreement between our calculation and the reported speeds in the literature supports our speculation that the tilt angle of the band is determined jointly by the propagation speed of the head and the advection speed of the streaks inside the bulk. However, what mechanism determines the advection speed of the streaks is still to be investigated. A quantitative study of the large-scale flow may give a hint to the advection of the streaks [9,27,29,30].

It should be noted that the two ends of turbulent bands may not exist in relatively small normal periodic domains or narrow tilted domains, therefore, seemingly our formulation of the tilt angle (Equation (1)) does not apply. In those cases, it is not clear what mechanism determines the tilt angle of turbulent bands. Our speculation is that the tilt angle may be indefinite and is strongly affected by the specific domain selection if the head does not exist. This might explain, for the same Reynolds number, why turbulent bands can exist in tilted domains with very different tilt angles [4,9,20] and why the nonlinear traveling wave solutions that Paranjape et al. [19] obtained can exist in a broad tilt-angle range from $20^{\circ }$ to $70^{\circ }$.

## 5. Discussion

The wave generation at the head, the tilt direction, the advection of the head, the streaks inside turbulent bands and the tilt angle of the band are discussed and investigated in this paper. The inflectional-instability argument of Xiao and Song [24] for the wave generation at the head and its potential relationship with the localized periodic-orbit theory of Kanazawa [7] are discussed. Based on the discussion, we propose that the tilt direction should probably be determined by the local inflectional spanwise velocity profile generated/introduced by the initial perturbation. The opposite tilt directions are rooted in the mirror symmetry of the spanwise velocity component. Besides, we measured the propagation speed of the head and the advection speed of the low-speed streaks in the bulk of turbulent bands at low Reynolds numbers up to Re=1050. We found that the head propagates at constant speeds of $c_{x}=0.85$ and $c_{z}=0.1$ (absolute value) at all Reynolds numbers investigated. The low-speed streaks are advected roughly at the speed of the bulk speed in the streamwise direction with a slight decreasing trend as the Reynolds number increases, and the spanwise advection speed is nearly constant at approximately 0.07. Prior studies measured the tilt angle by treating the band as a tilted object [6,9]; alternatively, we here propose that the tilt angle of turbulent bands should be determined by the kinematics of the head and the streaks generated at the head. Specifically, the tilt angle can be calculated using the relative speed between the streaks in the bulk and the head, and, at least for Re≲900, we obtained a good agreement with the experimental measurements of Paranjape [9]. We also speculate that the tilt angle of a band may be indefinite and system-dependent if the head does not exist as in narrow tilted domains and relatively small normal domains.

A few problems remain poorly understood and should be investigated in order to further understand the transition in channel flow.

- The sustaining mechanism of the wave-generating head. The formation and sustainment of the locally inflectional flow at the head, whether or not the head is locally self-sustained and the relationship between the head and the large-scale flow are still not clear. If the head is indeed locally self-sustained and independent of the bulk, as proposed by Kanazawa [7], how the flow can be locally excited to this periodic orbit is also not clear. This problem is relevant to the generation and control of turbulent bands at low Reynolds numbers.
- The mechanism underlying the advection speed of the head. Xiao and Song [24] speculated that the speeds are possibly determined by the speeds of the unstable waves resulting from the local inflectional instability. They reported a close spanwise speed of the most unstable wave for Re=750, which is about 0.1 and is close to the actual spanwise of the head (see Figure 2). However, the streamwise speed of the most unstable wave is roughly 0.55 (can be calculated from the eigenvalues and wavenumbers associated with the most unstable wave reported by them) and is significantly lower than the values shown in Figure 2, which is about 0.85. This discrepancy may be attributed to the over-simplification of the local mean flow at the head by temporal and spatial averaging in their linear stability analysis, as well as by the region selection for the averaging. A possibility to elucidate the mechanism underlying the advection speed is to investigate the speed of the periodic orbit of Kanazawa [7].
- The mechanism underlying the self-sustainment and advection speed of the streaks. Paranjape et al. [19] obtained exact traveling wave solutions that have some key characteristics of turbulent bands and identified the solutions as the precursors of turbulent bands. Further, for these solutions, they speculated that the streaks are sustained by the tilting effect of the large-scale flow, instead of the self-sustaining process of wall turbulence at high Reynolds numbers in which sinuous streaks break down, generating streamwise vortices, and are regenerated by streamwise vortices [31,32]. The same mechanism may also apply to turbulent bands. In our simulations, we indeed observed that streaks in the bulk are long-lived and move with a characteristic speed without a clear breakdown and regeneration. Duguet and Schlatter [27] described turbulent bands in plane shear flows as the advection of small-scale structures (streaks and vortices) by the large-scale flow, which also seems to suggest the important role of the advection by the large-scale flow in the sustainment of the streaks.
- The mechanism underlying the decay of streaks at the tail as well as the splitting and branching of turbulent bands. At relatively higher Reynolds numbers, a band may also nucleate a band with the opposite tilt direction [8,9]. The splitting scenario, at least partially, determines the flow pattern.

## 6. Materials and Methods

For solving the incompressible Navier–Stokes equations in channel geometry, we used our in-house code as described in [24,25], which adopts a high-order finite-difference method with a centered nine-point stencil in the wall-normal direction and Fourier-spectral method in the periodic streamwise and spanwise directions. Readers are referred to Openpipeflow [33] for details about the finite-difference scheme and the parallelization of the code. The Navier–Stokes equations were integrated using the method of Hugues and Randriamampianina [34], which adopts a second-order-accurate backward-differentiation scheme, combined with the Adamas–Bashforth scheme for the nonlinear term, for the temporal discretization and a projection method to impose the incompressibility condition. The time-step size was fixed at Δt=0.01 for the simulations presented in this paper, which was shown to be sufficiently small for the Reynolds number regime considered [4,6].

We adopted the method proposed by Song and Xiao [25] to generate turbulent bands in large domains. The method firstly derives a body force that is needed to maintain an inflectional velocity profile that bears a sufficiently strong instability. Given a target velocity profile U(y), the body force is derived as

(2) $$f=-\frac{1}{Re}\nabla^{2}U\left(y\right).$$

Then, the body force is multiplied by a localization factor such that the force is localized in the *x*-*z* plane. The size of the localization region should be comparable with the size of the head of a turbulent band and the forcing region is moved at the speed of $c_{z}=0.1$ (absolute value) and $c_{x}=0.85$ (see Figure 2). If the profile U(y) is sufficiently inflectional, the instability can generate sufficiently strong tilted waves (streaks and vortices) and trigger turbulent bands. Once triggered, the length of the band increases, and the force can be switched off after the band has sufficiently developed. The tilt direction of the band can be controlled by the signs of the spanwise component of U(y) and the moving speed $c_{z}$.

We measured the advection speed of the low-speed streaks inside the bulk using the Structural Similarity Index Measure (SSIM) method proposed by Wang et al. [28], which is commonly used in image processing to measure the similarity between two images. The SSIM index is defined as:

(3) $$SSIM\left(x,y\right)=l{\left(x,y\right)}^{\alpha }\cdot c{\left(x,y\right)}^{\beta }\cdot s{\left(x,y\right)}^{\gamma },$$

where *x* and *y* are one-dimensional vectors containing all the pixel values of the two images to be compared, respectively, and

(4) $$\begin{array}{c} l\left(x,y\right)=\frac{2\mu_{x}\mu_{y}+c1}{\mu_{x}^{2}+\mu_{y}^{2}+c1}, \end{array}$$

(5) $$\begin{array}{c} c\left(x,y\right)=\frac{2\sigma_{x}\sigma_{y}+c2}{\sigma_{x}^{2}+\sigma_{y}^{2}+c2}, \end{array}$$

and

(6) $$\begin{array}{c} s\left(x,y\right)=\frac{\sigma_{xy}+c3}{\sigma_{x}\sigma_{y}+c3}, \end{array}$$

measure the luminance, contrast and structural similarity, respectively. The exponents α>0, β>0 and γ>0 are used to tune the relative weight of respective factor, and here we set all of them to 1 according to the suggestion of Wang et al. [28]. In Equation (4), $\mu_{x}$ and $\mu_{y}$ denote the mean of *x* and *y*, respectively. In Equation (5), $\sigma_{x}$ and $\sigma_{y}$ denote the standard deviation of *x* and *y*, respectively. In Equation (6), $\sigma_{xy}$ is the covariance of *x* and *y*. Parameters $c1={\left(k_{1}L\right)}^{2}$ and $c2={\left(k_{2}L\right)}^{2}$, where k1 and k2 are set to 0.01 and 0.03, respectively, and *L* is the maximum of the pixel value, which is set to 255 for unit8 data and 1 for floating point data. In our calculation, the flow velocities, which are floating point data, were taken as the pixel value *x* and *y*. The parameter $c_{3}$ is set such that $c_{3}=c_{2}/2$ in practice according to the suggestion of Wang et al. [28]. Thus, we have

(7) $$SSIM\left(x,y\right)=\frac{\left(2u_{x}u_{y}+c_{1}\right)\left(2\sigma_{xy}+c_{2}\right)}{\left(u_{x}^{2}+u_{y}^{2}+c_{1}\right)\left(\sigma_{x}^{2}+\sigma_{y}^{2}+c_{2}\right)}.$$

The result is a value between −1 and 1, and the larger is the result, the higher is the similarity.

**Figure 10:**
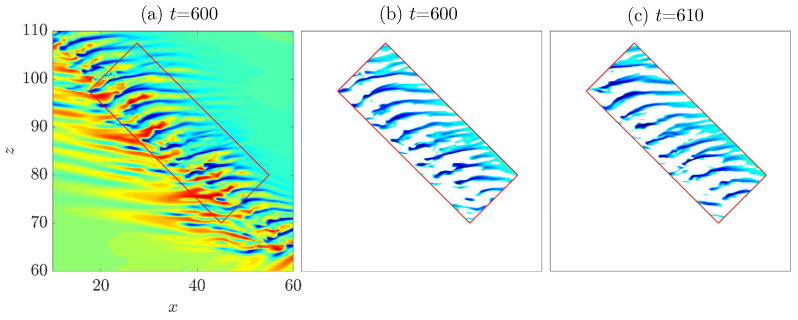
The selection of low-speed streaks for the advection speed calculation. The tilt angle of this rectangle is $45^{\circ }$, which is close to the tilt angle of the band. The area of the rectangle should be large enough to contain sufficient streaks and meanwhile reduce the influence of the tilt angle of the rectangle. (**a**) The contours of streamwise speedat y=−0.5 at t=600. The advection speed of the streaks in the region enclosed by the rectangle is calculated; (**b**,**c**) The filtered low-speed streaks at t=600 and t=610.

**Figure 11:**
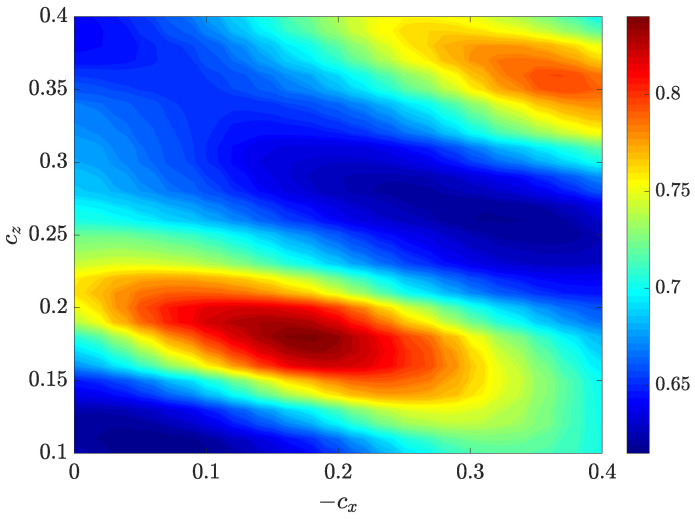
The contours of the SSIM index in the $c_{x}$-$c_{z}$ plane for the case shown in Figure 10.

Firstly, we take the streamwise velocities in the cut plane y=−0.5 from two different snapshots $s_{1}$ and $s_{2}$ that are separated in time by δt, after the tilt angle of the band has stopped changing considerably due to the initial transients. In the frame of reference co-moving with the head, i.e., moving with a streamwise speed of 0.85 and a spanwise speed of −0.1 (we considered a right-going band), the bulk of the band is located in a nearly fixed area (see Figure 10). Therefore, we set a rectangular area in which the data inside were considered for calculating the SSIM index. We set the data outside this area to zero so that we eliminated the influence of the data outside this area. Further, to highlight the low-speed streaks, only the streamwise velocities in this area that satisfies $u_{x}<0$ and $u_{x}^{2}>0.002$ were retained. Secondly, we shifted the data from $s_{2}$ inside the rectangular over the time separation δt with a streamwise speed $c_{x}$ and a spanwise speed $c_{z}$. The original data from $s_{1}$ and the shifted data from $s_{2}$ were used to calculate the SSIM index. Thus, for a given speed pair $\left(c_{x},c_{z}\right)$, there is a corresponding SSIM index. By varying the speed pair, the SSIM index will maximize with certain speeds, which we considered as the mean advection speeds of the streaks. The contours of the SSIM index in the $c_{x}$ and $c_{z}$ for Re=750 are shown in Figure 11.

Note that, in practice, we set $-c_{x}$ and $c_{z}$ to be between 0.1 and 0.4 (the band we considered is a right-going one; therefore, $c_{x}<0$ and $c_{z}>0$) because the actual speeds were estimated by eye to exist in this range, and note that the shift speeds are relative to the propagation of the head. Obtaining the contours of the SSIM index, we could estimate the advection speed of the streaks to be $c_{x}=-0.185$ and $c_{z}=0.18$, i.e., the location of the local peak at the left-bottom corner in Figure 11. It can be seen that there is another local peak at the right-top corner, which shows a lower SSIM index. That peak was reached when the $s_{2}$ data were shifted by more than one wave-length associated with the pattern of the low-speed streaks. The lower SSIM index of the top-right peak, i.e., lower similarity, indicates that the streaky pattern slowly change as it is advected in the bulk.

Note that the time separation δt between $s_{1}$ and $s_{2}$ cannot be too small, otherwise the streaks would have moved too little over the time separation and the speed measurement would be inaccurate. Likewise, it cannot be too large in which case the streaks would have moved by multiple wavelengths, which would also affect the speed calculation. In practice, estimated by eyes, a value between δt=10 and 15 is a good choice, and δt=10 in Figure 10 and Figure 11. In the end, by varying the time instant of $s_{1}$, we can obtain the average advection speed as a function of time and calculate the temporal average, which is plotted in Figure 7 (the speed of the head is added back in that figure).

## Figures and Tables

**Table 1:** The Reynolds number Re, domain size $L_{x}$ and $L_{z}$, number of wall-normal grid point *N* and the ratio between *h* and the grid spacing in *x* and *z* directions, Δx and Δz, respectively.

Re	$L_{x}\times L_{z}$	*N*	h/Δx	h/Δz
670	120 *h* × 120 *h*	72	4.3	6.4
750	120 *h* × 120 *h*	72	4.3	6.4
850	160 *h* × 160 *h*	72	4.8	6.4
950	160 *h* × 160 *h*	72	4.8	6.4
1050	240 *h* × 240 *h*	72	4.8	6.4
